# Acute Colonic Pseudo-Obstruction Following the Use of Dexmedetomidine

**DOI:** 10.7759/cureus.19465

**Published:** 2021-11-11

**Authors:** Hussam R Alkaissi, Aleksandr Khudyakov, Pooja Belligund

**Affiliations:** 1 Internal Medicine, State University of New York Downstate Medical Center, New York City, USA; 2 Pulmonology and Critical Care, Veterans Affairs New York Harbor Health Care (VA NYHHS), Brooklyn, USA

**Keywords:** alpha-2 agonists, ogilvie's syndrome, delirium tremens, acute colonic pseudo-obstruction, dexmedetomidine

## Abstract

Dexmedetomidine is a preferred agent for light sedation with minimal adverse effects. We report a case of acute colonic pseudo-obstruction following dexmedetomidine use in a patient with alcohol withdrawal. He was treated with benzodiazepines first to control the withdrawal symptoms, then escalated to dexmedetomidine once delirium tremens ensued. Later on, the patient developed abdominal distension and vomiting. Imaging showed dilated bowel loops and absence of peristalsis on ultrasound. Decompression with the nasogastric (NG) tube was done, with high output from the NG tube. Dexmedetomidine infusion was used twice, and once it was stopped, the NG tube output was reduced, with the resumption of gastrointestinal motility and improvement of the abdominal distension. Recent similar reports of functional intestinal obstruction following alpha-2 (α2) agonist use necessitate further studies of intestinal motility following dexmedetomidine use and awareness of the possible side effect of dexmedetomidine on intestinal motility.

## Introduction

Dexmedetomidine is an alpha-2 (α2) adrenergic receptor agonist used for sedation in mechanically ventilated patients. It is a preferred alternative to other sedatives, such as propofol, due to its easy and rapid reversibility yet with a better safety profile. It is also used in alcohol withdrawal syndrome patients resistant to benzodiazepines. Adverse effects are related to reduced sympathetic output, such as bradycardia and hypotension [1]. Cases of acute colonic pseudo-obstruction (ACPO) are reported with other α2 agonists, such as clonidine [2]. In this case, we describe a case of ACPO following dexmedetomidine use in a patient with an alcohol withdrawal syndrome.

## Case presentation

A 61-year-old male was admitted for acute alcohol intoxication and concern for impending withdrawal. At presentation, he was alert and oriented but tremulous and tachycardic to 110 beats per minute with normal blood pressure. Abdominal examination was normal. Clinical Institute Withdrawal Assessment Score (CIWA) of 6, indicating mild withdrawal requiring no medications. He had no nausea, vomiting, abdominal pain, or distension. Past medical history included hypertension, hyperlipidemia, alcohol abuse, and an episode of alcoholic pancreatitis. No cirrhosis, portal hypertension, or esophageal varices.

Laboratory findings were significant for an elevated alcohol level of 497 mg/dL, a microcytic anemia with a hemoglobin of 10.7 g/dL (13-15 g/dL), and mean corpuscular volume of 103 fL (80-100 fL), platelets of 111 x 10^3/μL (150-400 x 10^3/μL), aspartate aminotransferase 396 U/L (5-30 U/L), alanine aminotransferase 162 U/L (5-30 U/L), total bilirubin of 1.3 mg/dL (0.3-1 mg/dL) potassium of 3.4 mmol/L (3.5-5 mmol/L), and magnesium 1.7 mg/dL (1.7-2.2 mg/dL), lipase 149 U/L (10-150 U/L).

Initial supportive treatment included fluids with electrolyte repletion, thiamine, and folate infusion. The patient received a total of 6 mg of lorazepam (2 mg each) in the first 24 hours for CIWA scores of more than 8.

On day 3 of admission, the patient was agitated with visual and tactile hallucinations, tremulousness, and tachycardia ranging in 120s with a CIWA score of 12. The patient's lorazepam requirement went up to 16 mg over two hours. Chlordiazepoxide 50 mg every six hours was added to the regimen to control the withdrawal and was up-titrated to 100 mg every six hours. Due to concern for impending delirium tremens, the patient was admitted to the medical intensive care unit. On day 4 of his admission, the patient had become more agitated with uncontrollable shaking, raising blood pressure to 180/100 mmHg, suggestive of delirium tremens. Continuous infusion of dexmedetomidine was initiated at a rate of 0.2 μg/kg/hr and up-titrated to 1.5 μg/kg/hr with a dramatic resolution of withdrawal symptoms, and the patient became hemodynamically stable.

**Figure 1:**
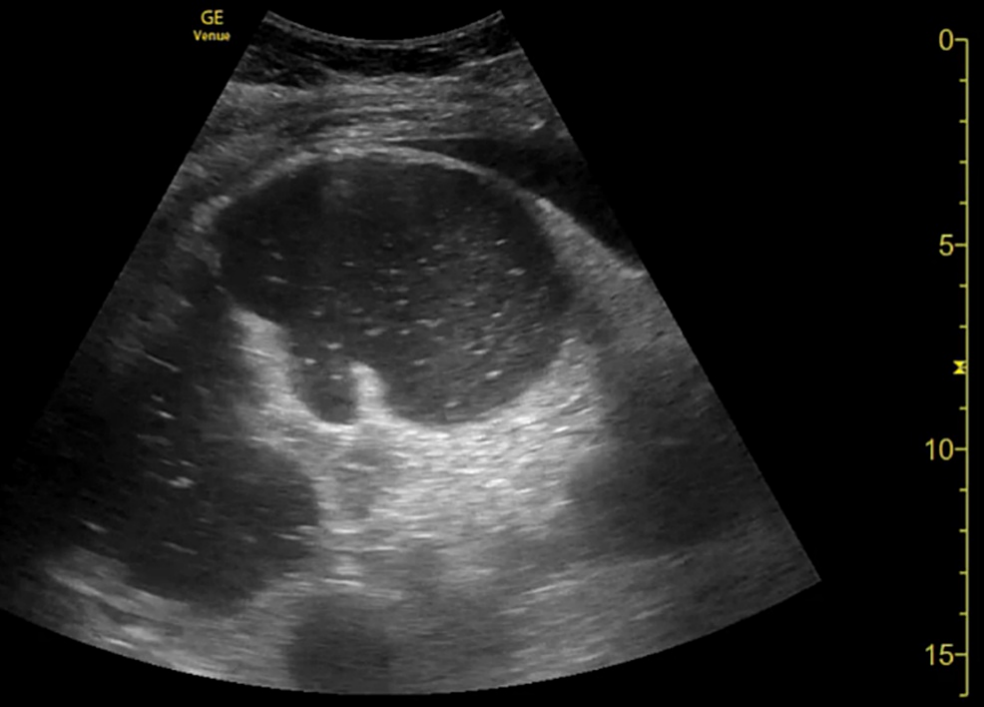
Point-of-care ultrasound showing dilated bowel loops in the right lower quadrant filled with anechoic secretions.

**Figure 2:**
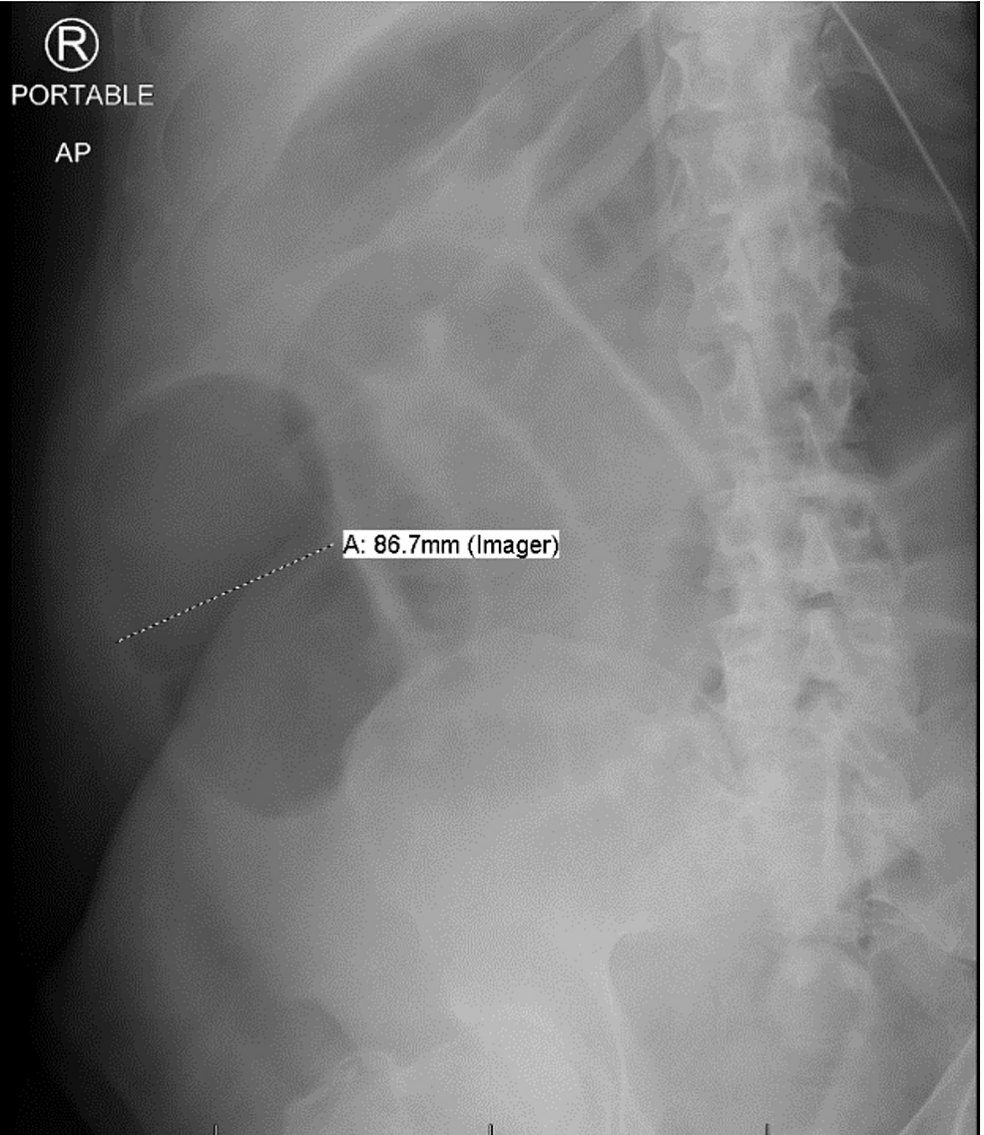
Portable abdominal x-ray demonstrating multiple dilated small bowel loops throughout the abdomen with the dilated cecum-ascending colon of more than 8 cm.

On the evening of his fifth day of admission, the patient was had voluminous, bilious, non-bloody vomiting with abdominal distension and diarrhea. A nasogastric (NG) tube was inserted with an estimated 1800 mL of output was noted over half an hour. Imaging was performed via bedside ultrasound (Figure 1) while waiting for an x-ray (Figure 2) confirmation of NG tube placement. Large, dilated bowel loops were noted on the ultrasound, confirmed by abdominal x-ray showing dilated small bowel loops (>3 cm) and dilated cecum of about 8.6 cm with no free air. Stool studies for *Clostridioides difficile *were negative. The patient developed hypoxic respiratory failure secondary to aspiration pneumonia and was intubated and treated with empiric piperacillin/tazobactam. He was switched from dexmedetomidine to fentanyl and midazolam for sedation. Computed tomography with oral contrast of the abdomen and pelvis showed oral contrast throughout the small bowel without evidence of mechanical bowel obstruction yet with bowel loops dilatation. Colonic dilatation was most pronounced within the transverse colon, measuring up to 6.7 cm. Laboratory data showed normal electrolytes, liver enzymes, and lipase.

The patient continued to have large volume output from the NG tube on day 7, significantly reduced by day 8 (Table 1). Extubation was planned, and the patient was transitioned back to dexmedetomidine 0.2 μg/hr/kg for sedation. A correlation between days-on-dexmedetomidine and NG tube output was noticed, and we suspected a pseudo-obstruction related to dexmedetomidine; thus, on day 10 of admission, dexmedetomidine was stopped, and the patient was successfully extubated, followed by reduction of NG tube output, resumption of intestinal motility, and reduction of abdominal distension.

**Table 1:** Illustration of the patient's increasing NG tube output after resuming dexmedetomidine on day 8. Checkmark indicating days on dexmedetomidine

Days	3	4	5	6	7	8	9	10	11
NG Tube output	NA	NA	1,300	1,000	750	350	1,350	0	Removed
Dexmedetomidine	✔	✔	✔	✔		✔	✔		

On day 11, NG tube was significantly reduced and was subsequently removed. The patient had an uneventful remainder of his hospital course and was discharged afterward.

## Discussion

ACPO was first described in 1948 by Sir Ogilvie in two patients where surgical exploration for intestinal obstruction revealed no mechanical etiology but rather a malignant infiltration of para-aortic ganglia, implicating the autonomic nervous system for a non-mechanical, functional obstruction [3]. The estimated annual incidence of ACPO is about 1:1,000, and mortality of about 7.7%. Early recognition and proper management are of paramount importance [4]. Management includes decompression (NG tube, colonoscopic decompression), medical (parasympathomimetic), and surgical after the failure of medical management [5,6].

To understand ACPO pathophysiology, one must first examine the physiology of colonic motility. Colonic motility is governed by pacemaker signals from interstitial cells of Cajal, with modulation through the autonomic nervous system and colonic arc reflex. Any disruption of those factors may result in ACPO. For example, ACPO may occur in old age or neurodegenerative disorders where there is loss of Cajal cells or inactivation of colonic arc reflex. Similarly, autonomic imbalance with increased sympathetic overflow or reduced parasympathetic control may lead to an atonic area in the colon with proximal dilatation. Such imbalance is seen with sympathetic agonists and parasympathetic antagonists [6].

Here, we report a case of ACPO following exposure to dexmedetomidine. Dexmedetomidine is an α2-adrenergic receptor agonist, first described in animal models in 1986 [7]. It was approved by the American Food and Drug Administration (FDA) in 2015 and European Medicines Agency (EMA) in 2020 as a safe option for light sedation. It is widely used in ICU settings for mechanical ventilation as an alternative for propofol with a similar safety profile [8-10]. α2-adrenergic receptors function as negative feedback controllers of the sympathetic nervous system. Upon their activation, α2 receptors reduce the sympathetic output. Dexmedetomidine has about 1,600:1 affinity for α2:α1 receptor binding, which explains its sedative effect by preferentially binding to α2 receptors in locus ceruleus, thus reducing the norepinephrine released from locus ceruleus [11,12]. α2 receptors are also present in the vagus nerve and enteric neurons, and their activation leads to a reduction of acetylcholine release [13].

Dexmedetomidine seems to have a dichotomous effect on intestinal motility. In one study, dexmedetomidine at a low dose of 0.4 µg/kg/hr, infused during laparoscopic surgery, reduced time to first flatus postoperatively, and reduced related ileus [14]. This effect is likely due to the sympatholytic, central effect of dexmedetomidine on locus ceruleus sympathetic output. However, at higher doses, dexmedetomidine slowed gastrointestinal motility significantly. In a study of 12 healthy subjects, dexmedetomidine at 0.7 µg/kg/hr for about three hours markedly slow gastric emptying and orocecal transit time compared to morphine and placebo [15]. A similar in vivo study was done in endotoxemic mice, where dexmedetomidine resulted in the slowing of colonic transit time [16]. This effect is likely to be peripheral acting on the vagus nerve and enteric nervous system by reducing acetylcholine release, as shown in animal studies. Guinea pigs' intestines were taken out and immersed in saline to stimulate peristalsis. When dexmedetomidine was added, a clear inhibition of saline-induced peristaltic activity, pointing to a direct peripheral effect on the intestine [13]. To put it all together, dexmedetomidine at low doses and a brief exposure time causes a reduction of sympathetic output and promotes intestinal motility. On the other hand, higher doses and prolonged exposure can cause a reduction of parasympathetic activity and slow peristalsis and, in extreme cases, can lead to ACPO.

Cases of ACPO have been reported with other α2 agonists, such as clonidine; ACPO occurred after two to five days after exposure to a high dose of clonidine in these cases [2,17]. One of them was in a patient with delirium tremens as well. ACPO following amitraz exposure, an insecticide with α2 agonist activity, has also been reported [18].

In 2019, Pérez-Lara et al. reported a similar case of dexmedetomidine-induced ACPO [19]. Awareness of that report leads to early suspicion that dexmedetomidine might be the culprit, primarily when the second exposure was associated with increased NG tube output. Early recognition lead to early discontinuation of dexmedetomidine, resumption of intestinal peristalsis, and less severe outcome overall without need for surgical intervention.

Other factors including critical illness, electrolytes imbalance, and medications may contribute to the clinical picture as well. Our patient has been exposed to fentanyl and several benzodiazepines used to control alcohol withdrawal symptoms. One may argue that any of those medications might be responsible for the ACPO or potentiate dexmedetomidine's effect. Fentanyl, an opioid, is known to slow intestinal motility at much lower rates than other opioids such as morphine. The patient was mildly hypokalemia on day 1 with potassium of 3.4 mEq/L, which was corrected on the same day. Electrolytes were stable throughout the rest of the hospitalization, thus making it less likely as a cause of the ACPO. Dexmedetomidine was compared to morphine and was significantly more paralyzing to intestinal motility and gastric emptying than morphine, making it much more likely as the cause of ACPO [15]. Another point that strongly suggests that dexmedetomidine was the cause of the ACPO is the temporal association. NG tube output was reduced one day after discontinuing dexmedetomidine, then bounced up again one day after reintroducing it. Critical illness is also known to cause upper and lower gastrointestinal dysmotility, which might have been a catalyst in the manifestation of ACPO once dexmedetomidine was introduced [20]. In retrospect, this served as a challenge test where the reintroduction of the suspected medication results in recurrence or worsening of the adverse reaction. Similarly, the patient's electrolytes were repleted since day 1 and remained stable, pointing away from electrolyte disturbance as a cause of the ileus. Our case scores 10 out of 12 points using Naranjo's adverse drug reaction probability scale, marking it a definite adverse reaction to dexmedetomidine.

## Conclusions

Dexmedetomidine is an attractive new agent for light sedation with few adverse effects. The most common adverse effects are related to sympatholytic effects, such as bradycardia and hypotension. Gastrointestinal effects are less well studied but they may include slowing of gastrointestinal motility with functional obstruction. Awareness of such adverse effect profiles may result in early identification and early discontinuation of dexmedetomidine leading to a rapid resumption of intestinal motility.
